# Engineering Meteorological Features to Select Stress Tolerant Hybrids in Maize

**DOI:** 10.1038/s41598-020-60366-y

**Published:** 2020-02-25

**Authors:** Gordan Mimić, Sanja Brdar, Milica Brkić, Marko Panić, Oskar Marko, Vladimir Crnojević

**Affiliations:** University of Novi Sad, BioSense Institute, Novi Sad, 21000 Serbia

**Keywords:** Environmental sciences, Climate-change mitigation

## Abstract

In this study we used meteorological parameters and predictive modelling interpreted by model explanation to develop stress metrics that indicate the presence of drought and heat stress at the specific environment. We started from the extreme temperature and precipitation indices, modified some of them and introduced additional drought indices relevant to the analysis. Based on maize’s sensitivity to stress, the growing season was divided into four stages. The features were calculated throughout the growing season and split in two groups, one for the drought and the other for heat stress. Generated meteorological features were combined with soil features and fed to random forest regression model for the yield prediction. Model explanation gave us the contribution of features to yield decrease, from which we estimated total amount of stress at the environments, which represents new environmental index. Using this index we ranked the environments according to the level of stress. More than 2400 hybrids were tested across the environments where they were grown and based on the yield stability they were marked as either tolerant or susceptible to heat, drought or combined heat and drought stress. Presented methodology and results were produced within the Syngenta Crop Challenge 2019.

## Introduction

Maize (*Zea mays* L.) is one of the world’s most important crops. Every year, breeders create a large number of experimental hybrids and measure their performance across different environments to select hybrids with the highest yield. The best hybrids have been identified by trial and error. However, this process can take many years^1^. Research question of Syngenta Crop Challenge in Analytics 2019 was the following: Can environmental data be aggregated into useful metrics representing stresses encountered by maize throughout a growing season? Can these metrics be used to discriminate between hybrids tolerant and susceptible to the stresses they represent?

Due to climate change, extreme weather events, such as spells of very high temperature, droughts or torrential rains, are becoming more frequent and sever^2^. Very often, these events are causing crop damage and increasing cost for the producers, especially in the USA^3^. Altered weather patterns in a specific region are changing temperature and precipitation regimes during the year^4^. Under the influence of solar radiation, temperature and precipitation, as the main drivers, crop grows and develops. Extreme weather in the growing season results in environmental stress, affecting plant development.

Soil water deficits accompanied by excessively high temperatures are already indicated as the most probable yield limiting factor in maize production in the USA^5^. Previous studies showed that daily maximum temperature greater than approximately 30 °C limits maize yields^6,7^. Statistical studies of rainfed maize yields indicated two clear features: a strong negative yield response to accumulation of temperatures above 30 °C or extreme degree days (EDD) and a relatively weak response to seasonal rainfall^8^. The dominant effect of EDD is increased vapour pressure deficit which drives faster transpiration rates in plants.

Drought is the most important environmental stress that limits crop yield^9^, especially in a global warming world. Main indicator of drought is the lack of precipitation during some period of time and if that is happening in the growing season, it can result in abiotic stress since plants would not have enough water for the proper development. Frequently used extreme precipitation index indicates maximum number of consecutive dry days with precipitation  <  1 mm^10^. Another way to do that, i.e. indicate conditions with drought, is to calculate vapour pressure deficit (VPD)^11^. While in the mentioned paper the sum of VPD was calculated during the growing season, the average VPD can be calculated due to different growing season length in different environments.

Maize growth stages and vulnerability of the plant to drought and heat stress during the specific stage can be found in the literature^12^. Drought and heat stress at flowering can cause asynchrony in the tasselling and silking of maize^13^. It is clearly stated that the plant is sensitive to dry conditions during the pollination and the 4-weeks period after the tasseling is very critical^14^. Also, the stress during the grain filling period can affect yield at the end of the season^15^. Here we roughly divided growing season length (GSL) into four stages: 1) from planting date up to the tasseling (VT) approximately at 30% of GSL, 2) from tasseling to the end of blister stage (R2) approximately at 50% of GSL, 3) from milk stage (R3) to physiological maturity (R6) approximately at 75% of GSL, and 4) from physiological maturity to harvesting date. Percentages were estimated from the Pannar website^16^. Approximate duration of the maize growth stages was given for the average growing season length of 160 days from emergence to harvest, to which we added another 10 days from planting to emergence^17^.

In order to answer the research question we engineered meteorological features to represent environmental stress throughout the growing season, developed the model for yield prediction, explained the contribution of the features to yield decrease and classified the hybrids based on their performance in the environments with different level of stress.

## Material and Methods

### Data

For crop challenge 2019 Syngenta provided two datasets, performance and weather, related to their experimental sites with diverse environmental conditions across United States.

Performance dataset encompassed 387,427 samples, which contained hybrid ID, yield (quintiles/hectare), environment ID as the identifier for the tested location (latitude, longitude) and year, planting date, harvesting date, irrigation and soil conditions, provided from 2008 to 2017. The soil variables included the percentage of sand, clay and silt, pH in soil, organic matter (OM), cation-exchange capacity (CEC), available water content (AWC) and saturated hydraulic conductivity of soil (KSAT). The number of hybrids in the experiment was 2,452 and the number of environments was 1,560.

Weather dataset consisted of the daily values of day length (seconds), solar radiation (*W*/*m*^2^), maximum temperature (°*C*), minimum temperature (°*C*), precipitation (mm), vapour pressure (Pa) and snow water equivalent (*kg*/*m*^2^) for all environments. After removing samples with unknown irrigation ( ≈ 2%), removing some illogical samples where harvest date was before the planting date and averaging yield for the same hybrid and environment, remaining dataset consisted of 122,881 samples.

### Methods

**Figure 1:**
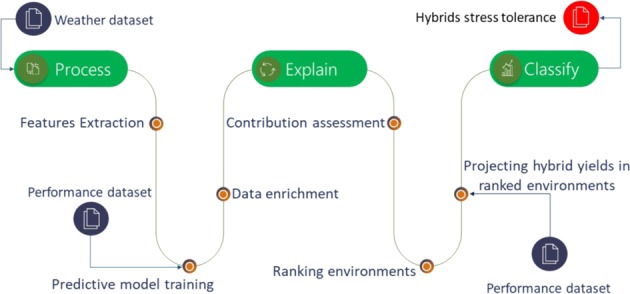
Processing steps diagram.

We developed analytical workflow that includes modules for processing of performance and weather data, learning predictive models, calculating stress metrics and decision making on whether hybrid is stress tolerant or not. The pipeline of methodology used in this study is presented in a diagram in Fig. 1 and all steps are further described in the following subsections.

The analytical workflow was developed using Python through the IDE PyChram^18^ and the suitable libraries such as pandas, numpy, sklearn, scipy, matplotlib and cartopy^19^.

### Engineering meteorological features

From weather dataset we used the values of given weather parameters for each environment and sort them in the proper order. In climatology, indices of extreme temperatures commonly used in the analysis are tropical days (annual count when daily maximum temperature > 30 °C) and tropical nights (annual count when daily minimum temperature > 20 °C)^10^. Here, we modified those indices and observed maximum number of consecutive tropical days and maximum number of consecutive tropical nights within the growing season, since these periods can result in heat stress in the plants and reduce the yield at the end of the season^20^. Also, in this study we used simple precipitation sum in different stages of the growing season, to distinguish between dry and wet conditions in different environments. World Meteorological Organization (WMO) listed several drought indicators and indices suitable for the analysis, but most of them need long term statistics, to be calculated. One of the indicators is hydro-thermal coefficient of Selyaninov (HTC), which is found to be more convenient for the growing season^21^. HTC presents the ratio between precipitation sum during the specific period of time and evapotranspiration, which is estimated as 1/10 of the sum of average temperatures > 10 °C, during the same period of time. Values between 0 and 1 correspond to dry conditions.

**Figure 2:**
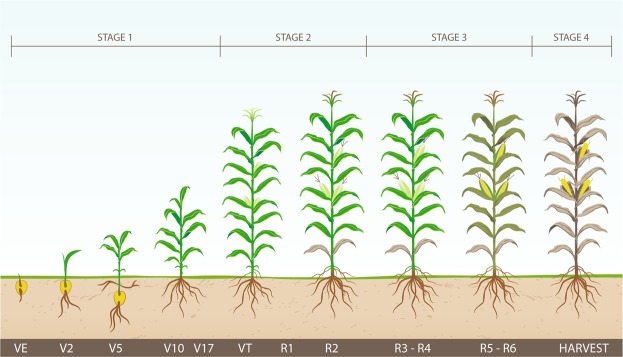
Maize growth stages based on plant’s vulnerability to stress.

For each environment, we calculated meteorological features which indicate heat and drought stress, in four stages during the growing season (Figure 2). Heat stress was represented by the following features: extreme degree days (EDD), maximum number of consecutive tropical days (CTD) and maximum number of consecutive tropical nights (CTN), while drought stress was presented with: maximum number of consecutive dry days (CDD), precipitation sum (PS) (mm), average vapour pressure deficit (VPD) (mbar) and hydro-thermal coefficient of Selyaninov (HTC). The exception was made with HTC because it could not be calculated in all the environments for the stages 1 and 4, due to the low average temperatures and hence, these two features were not considered in the analysis. Different level of irrigation was distributed throughout the environments in the following way: no irrigation 77.96%, very light irrigation 0.16%, light irrigation 1.77% and normal irrigation 22.11%. Furthermore, we used soil and meteorological features to run the yield prediction model.

### Predictive modelling of yield

The predictive model of the yield was trained on the environmental level, based on the extracted meteorological and available soil features, with mean yield of each environment as the target variable. We calculated the mean yield for each of the 1,492 environments left after preprocessing step.

We used random forest (RF) as the regression model, which has been proven to work well in agricultural use-cases^22–24^. It is an ensemble learning method based on a large number of decision trees, whose predictions are aggregated at the output level. Passing through trees is very fast since the computer only needs to go through a number of simple “if” statements. RF can deal with non-linear data and categorical features. It is robust to outliers and does not have issues with overfitting^25^. A total of 100 trees in the forest were considered. K-Folds cross-validation was used for performance evaluation. This means that the dataset was split into k = 10 folds, each of which served as the test set in one iteration and as a part of the training set in 9 others. Model results were evaluated with R^2^ score (coefficient of determination), root mean square error (RMSE) and Pearson correlation coefficient^26^.

### Data enrichment with Gaussian mixture model

**Figure 3:**
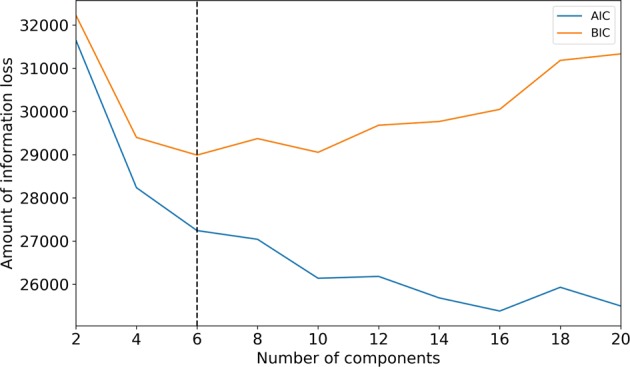
Estimation of the number of components (6) used in the Gaussian mixture model based on Akaike information criterion (AIC) and Bayesian information criterion (BIC) minimum values.

To extract knowledge from the predictive model we aimed at utilizing general model explanation method that can unveil black box classifiers. Such method relies on data sampling procedures, running model and quantifying changes in predictions. For better sampling we exploited Gaussian mixture model (GMM) to enrich the data used for training the predictive model. GMM, defined as a convex combination of Gaussian densities, was used for modelling of the probability density function (PDF) of our dataset ${\bf{X}}=\{{{\bf{x}}}_{n}| {{\bf{x}}}_{n}\in {{\mathbb{R}}}^{d},n=1,2,\ldots ,N\}$ where *d* is the dimension of each sample and *N* corresponds to the number of given samples. For each of *N* = 1492 environments we had eight features which described soil characteristics and 26 features for weather conditions, resulting in *d* = 34 features per each environment. We first reduced the dimensionality of our dataset using Principal Component Analysis (PCA) by keeping as many components as needed such that 99% of variance in the data is explained. In this way we projected our data, represented as matrix **X**^*N*×*d*^, onto a 9-dimensional subspace **W** ⊂ **X**, (**W**^*N*×*k*^ with *k* = 9) in which we further applied GMM training procedure to estimate the density of our dataset. Therefore, the PDF for each environment **w** (row in the matrix **W**^*N*×*k*^) was modeled as

1 $$p({\bf{w}})=\mathop{\sum }\limits_{j=1}^{M}{\pi }_{j}{\mathcal{N}}({\bf{w}};{{\boldsymbol{\mu }}}_{j},{\Sigma }_{j})$$

where *M* is the number of Gaussian components denoted with ${\mathcal{N}}({\bf{w}};{{\boldsymbol{\mu }}}_{j},{\Sigma }_{j})$, where ***μ*** = {***μ***_*j*_} stands for the centres of the components and corresponding covariance matrices *Σ* = {*Σ*_*j*_}, while ***π*** = {*π*_*j*_} are the mixing coefficients (weights). The first problem that can arise in GMM training is related to the number of components *M*. The choice of *M* is a matter of trade-off between under- and over-fitting. Too small *M* will not account for the specifics in the dataset, while too large *M* will fit too closely to the data and loose the GMM’s ability to generalize. The second problem is that estimated covariance matrices might become singular during the training procedure. In order to overcome these issues we opted for a variational Bayesian GMM (VB-GMM) procedure^27^. VB-GMM allowed us to impose the prior to the model parameter ***π*** with a Dirichlet process. In practice, Dirichlet process inference algorithm is approximated and uses a truncated distribution with a fixed maximum number of components (called the Stick-breaking representation^28^). Since VB-GMM determined the number of components *M* by setting some weights of the initial number of components very close to zero, we still needed this initial guess for the number of components. This was solved by fitting the expectation maximization GMM model without prior on our dataset by changing the number of components from two to 20 and calculating the Akaike information criterion (AIC) and Bayesian information criterion (BIC). Both information criteria estimate the relative amount of information lost by a given model, the less information a model loses, the higher the quality of that model. According to the obtained curves for calculated criteria we got two numbers of possible initial number of components in the model, one for which AIC criterion reached minimum value and other for which BIC criterion reached minimum value. We chose minimum of those two numbers (six in our experiments) in order to preserve model from overfitting (Fig. 3). With this number we started VB-GMM training procedure and estimated p.d.f. of the given dataset. Once we trained our model we generated samples in lower-dimensional subspace, applied inverse transformation to transform them back to original space and such that used in further analysis.

### Model explanation

Model explanation was used to get a better insight in the contribution of the features in yield prediction^29–31^. The method that was used is general and could be applied to any classification or regression model to explain how features contribute to predictions. For every feature, we divided the range of the feature values from minimum to maximum into 10 bands, bounded by 11 equidistant values. Then, we examined the contribution of each of these feature’s values to the yield prediction. The GMM trained on the dataset was used to generate 10,000 surrogate samples, where each sample was given in the form of a feature vector. We then randomly chose 500 samples from the enriched dataset and altered the value of the feature that we wanted to test. We altered the value of this feature in all 500 samples to the desired value and calculated the difference between the predicted yield of original and altered samples. This analysis resulted in 500 yield differences, for which we calculated the mean and standard deviation. The procedure was repeated for all selected values and all features from the dataset.

### Ranking the environments

The next step in the research was to relate the performance of hybrids to different levels of heat and drought stress. But first, as the yield prediction model was applied on the environmental level, we needed to define stress metrics that will allow us to rank the environments according to heat and drought stress. To quantify how much stress was present in each environment we utilized previously calculated contributions of features. Feature value observed in the environment was approximated by the nearest from available 11 equidistant values ranging from feature minimum to maximum and corresponding contribution was derived. With this approach we were able to assign the contribution of each feature value to yield prediction. Next, we summed up the contributions of all features, separately for heat and drought, and proclaimed these values as the total amount of heat/drought stress in a particular environment. As a result, the environments were ranked based on the total amount of heat or drought stress present in them. Now the interaction of hybrids and environments could be translated to a new space defined by the environment stress level and hybrid yield. Hybrid performances were thus projected onto this space to further examine their tolerance to stress.

### Yield stability

To characterize the response of a hybrid to stress across all the environments where it was grown, linear regression was performed between the yield and the level of stress at the environments, for heat and drought separately. On the other hand, multiple linear regression was used to examine the combined effect of both heat and drought stress. The slope of the regression was used as a measure of yield adaptability^32^. Slopes  > 1 indicated a greater change in yield with changing environmental conditions, and slopes  < 1 indicated more stable yields across a range of environments, classifying the hybrid as stress tolerant. In multiple linear regression both slopes for heat stress and drought stress had to be  < 1 to classify the hybrid as tolerant to combined heat and drought stress. Samples where normal irrigation was applied were excluded from the stability analysis since it was not relevant to consider the yield at the certain environment if the water deficiency was overcome and the stress was reduced^33^.

## Results

**Figure 4:**
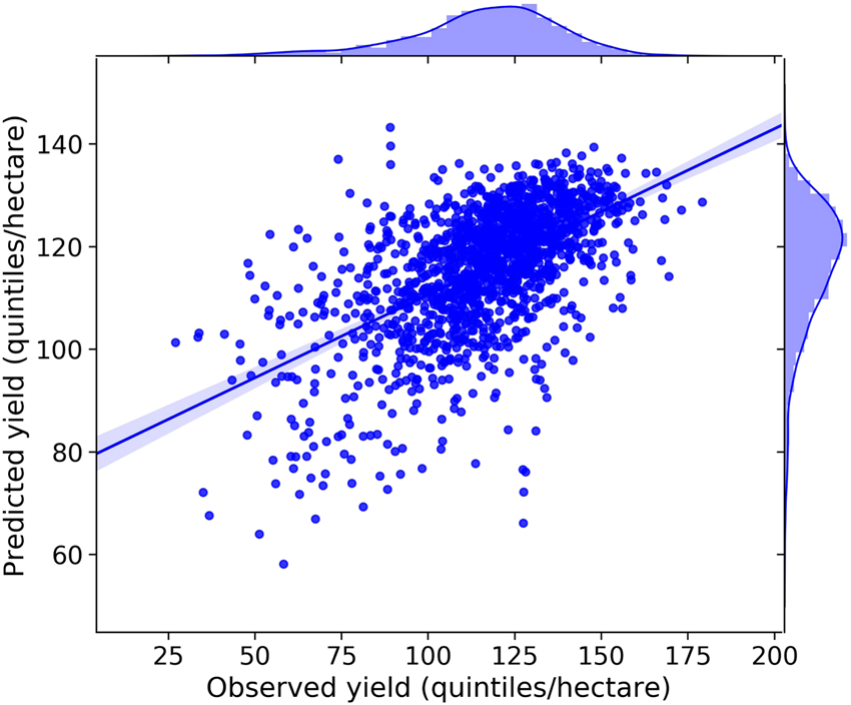
Scatter plot between observed and predicted yield.

The results of RF regressor were evaluated by comparing observed and predicted yield. Obtained results with R^2^ score = 0.33, RMSE = 18.0 quintiles/hectare and Pearson correlation coefficient = 0.58 showed moderate correlation between them (Fig. 4). After leaving the samples where normal irrigation was applied out of the analysis, we got the following results R^2^ score = 0.47, RMSE = 15.68 quintiles/hectare and Pearson correlation coefficient = 0.69, which proved that there is an impact of irrigation on the yield. However, we kept all the samples in further analysis for the following reasons: a) not to reduce dataset for 22.11%, b) to examine contribution of heat and drought stress features to the yield across all environments and c) to examine performance of the hybrids resistant to all stresses, in optimal conditions, where normal irrigation was applied.

**Figure 5:**
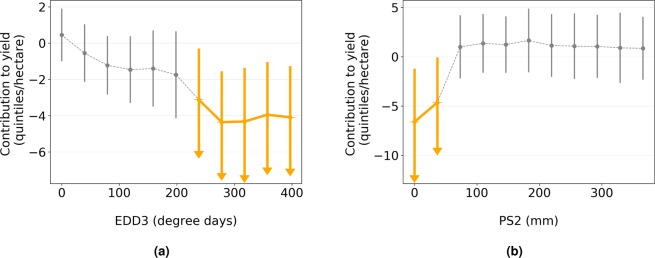
Contribution to the yield for EDD3 (**a**) and PS2 (**b**) obtained through the model explanation. Circles represent mean values, bars denote the standard deviation while arrows stand for the significance.

The main output of model explanation was the contribution of each feature to the yield prediction. The contribution was expressed as the mean value over 500 iterations. Example of the contribution of two features representing stress metrics EDD3 (heat) and PS2 (drought) is presented in Fig. 5. The standard deviation was summed up with the mean value and if the result was less then zero, the contribution was proclaimed as significant and shown with an arrow in the given figure. In the previous example, the figure on the left shows that high values of EDD3 have a negative effect on the yield, while the figure on the right shows that it is the low values of PS2 that affect the yield negatively.

The mean contributions of all features, obtained through the model explanation, had both positive and negative values. We provide an example in Table 1 for the environment Env_578. Since we designed the features to reflect hot and dry conditions, most of the values were negative, and some of them were quite low. Summation of all stress metrics corresponded to different levels of yield decrease, thus justifying the taken approach and the designed stress metrics. In the given example, the total amount of heat stress was −2.47 indicating that mean yield decreased for -2.47 quintiles/hectare at the specific environment due to the heat stress, while the total amount of drought stress was -13.15 indicating even higher yield loss in quintiles/hectare due to drought stress.

**Table 1:** Contribution of heat metrics (quintiles/hectare) and drought metrics (quintiles/hectare) to the yield for the environment Env_578.

Stress metrics	Stage 1	Stage 2	Stage 3	Stage 4
Heat metrics	EDD1	EDD2	EDD3	EDD4
	− 0.72	− 0.22	− 0.74	− 0.15
	CTD1	CTD2	CTD3	CTD4
	− 0.53	0.27	− 0.27	0.11
	CTN1	CTN2	CTN3	CTN4
	− 0.22	0.07	0.24	− 0.31
Drought metrics	PS1	PS2	PS3	PS4
	− 0.73	1.37	− 0.03	− 2.72
	CDD1	CDD2	CDD3	CDD4
	0.26	− 0.09	− 0.67	− 1.96
	VPD1	VPD2	VPD3	VPD4
	0.18	− 2.01	− 6.31	1.75
		HTC2	HTC3	
		− 1.76	− 0.43	

**Figure 6:**
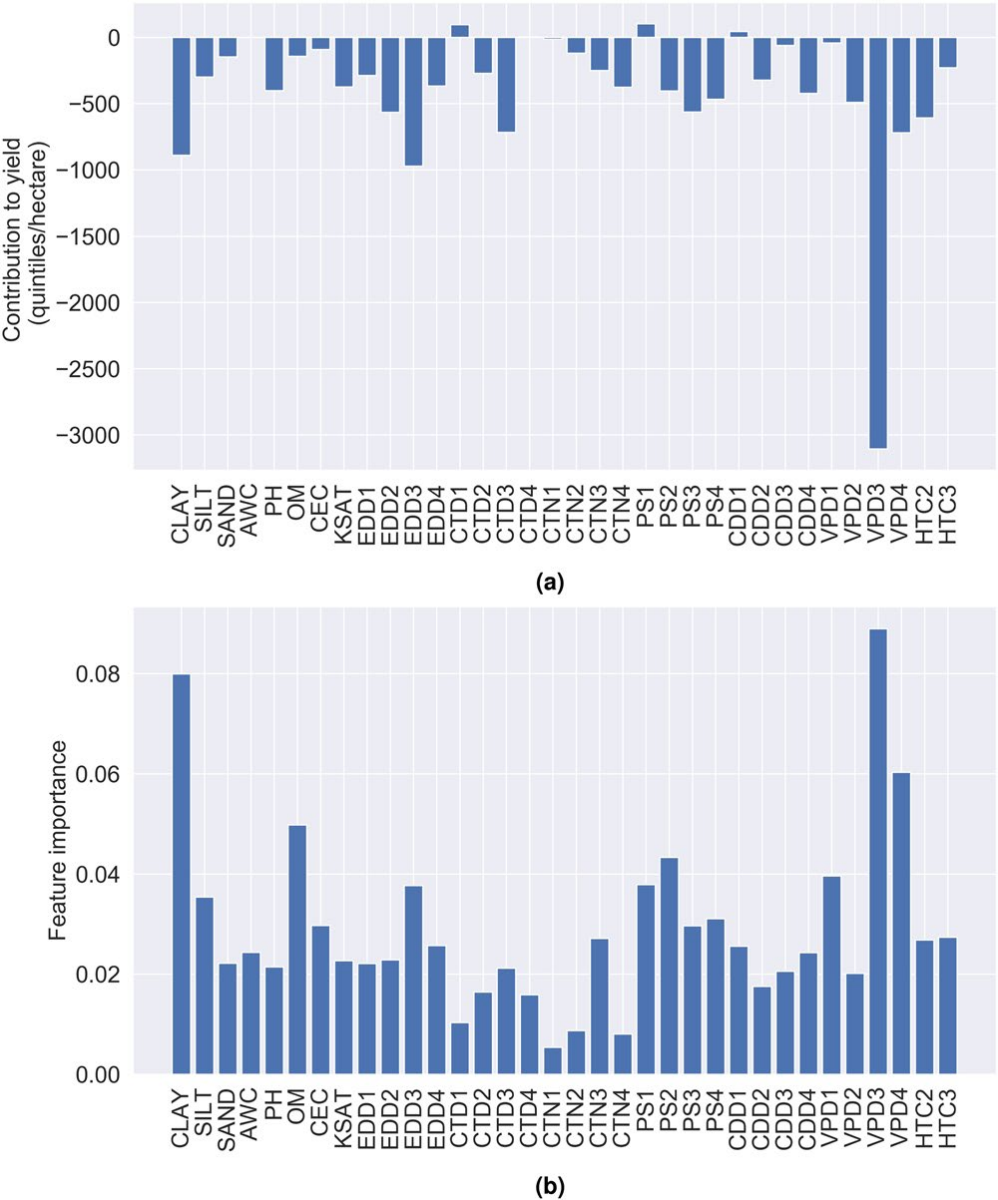
Feature contribution to yield (quintiles/hectare) summed up across all environments (**a**) and feature importance score from the regression model (**b**).

After we summed up the contribution of each feature across all environments the results showed which contributed the most to the yield (Fig. 6(a)). The highest contribution had vapour pressure deficit in stage 3. Along with it, other features from stage 3, such as extreme degree days, maximum number of consecutive tropical days and precipitation sum, contributed with high rate to the yield. Once again, this confirmed the fact that maize is very sensitive to heat and drought stress in the grain filling period. Regarding the soil features, two most dominant were percentage of clay and pH value. From the other hand, feature importance score, indicated from the regression model, showed strong consistency with feature contribution. The most important feature for the data-driven model was vapour pressure deficit in stage 3, followed with the percentage of clay (Fig. 6(b)). Some authors discussed that soil with higher clay content also have high transpiration and crop evapotranspiration rate^34^ and that lower yields can be realized in dry conditions on the soil with higher clay content compared to the management zones with lower clay content^35^ while this phenomenon appears reversed in wet conditions. Texture classes between 10–30% of clay content had air and moisture regimes that are optimal for healthy maize production (see Supplementary Fig. S1).

**Figure 7:**
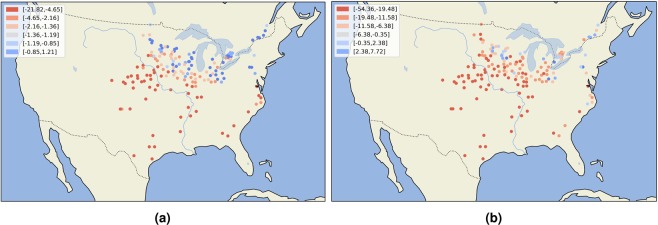
Environments ranked by the total amount of heat stress (**a**) and drought stress (**b**) for the year 2012.

Environments were ranked based on the total amount of stress. Example of ranking in one season is given in Fig. 7 for the year 2012, where lower values indicate higher level of stress (for all the years see Supplementary Figs. S2 and S3). Colour palette from blue to red further indicates lower to higher stress levels. Right panel represents moderate to sever drought in the US Midwest, which was actually observed in the growing season 2012^36^. These results can be considered as the proof of concept. The drought of 2012 was an agricultural disaster in the United States^37^.

It was possible that a hybrid was grown only in environments with low level of stress or without any stress at all. In that case it would be impossible to do proper classification. To make the criterion for the selection of stress tolerant hybrids more rigorous, we considered environments that had values of heat stress and drought stress  <  10th percentile, representing extreme conditions. If the hybrid was grown only in the environments with stress values  >  10th percentile it was not taken into consideration since it was never grown in extreme conditions. That hybrid was assigned to the group of hybrids non-tolerant to stress.

**Figure 8:**
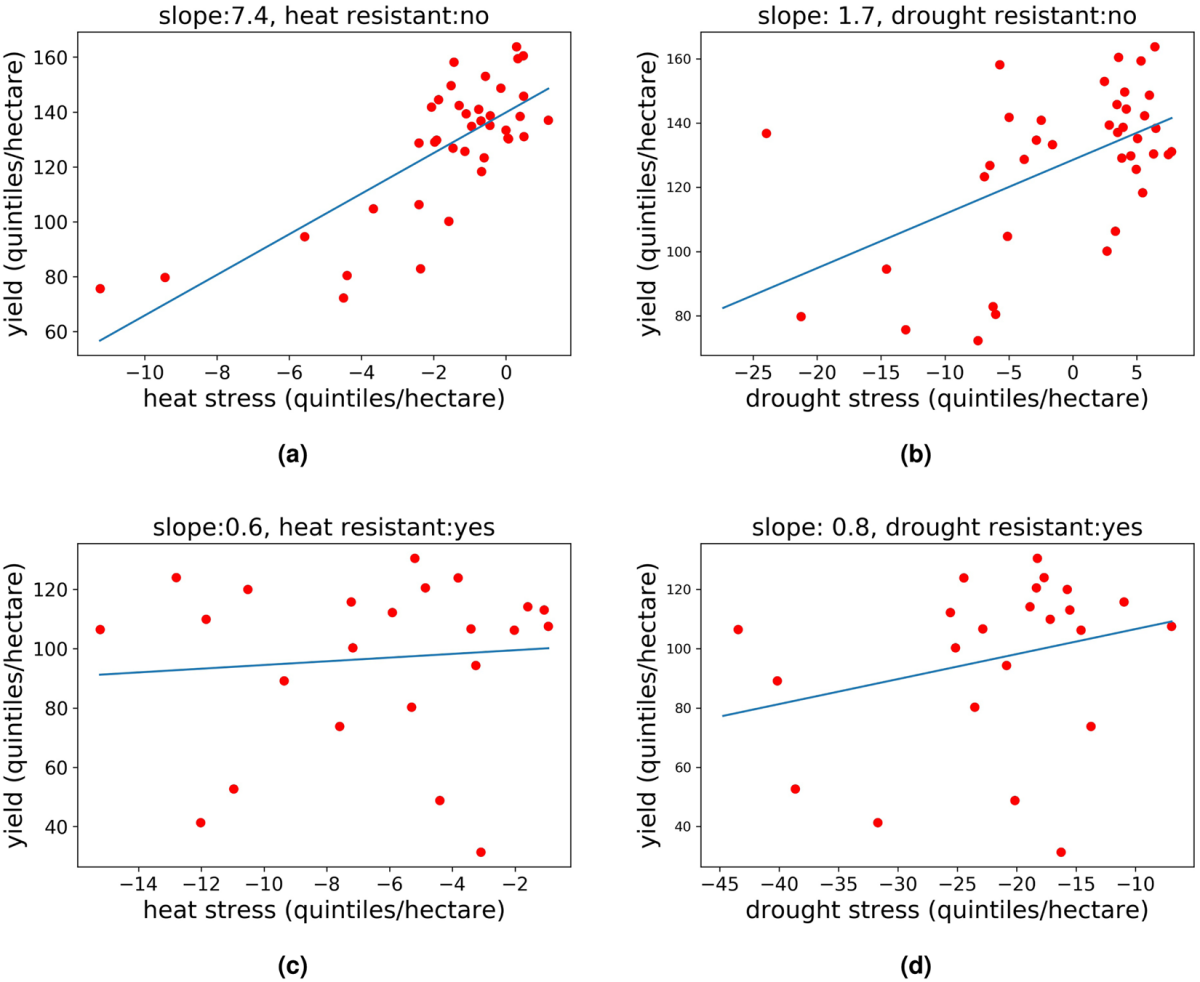
Performance of hybrid H1082 in different environments with heat stresses (**a**) and drought stresses (**b**) and hybrid H2058 in different environments with heat stresses (**c**) and drought stresses (**d**).

In Fig. 8 performance of two hybrids across environments with different level of heat and drought stress was given. Results obtained by the presented methodology address that the hybrid H1082 is non-tolerant to heat and also to drought (Fig. 8(a,b)), while hybrid H2058 is tolerant to both heat and drought (Fig. 8(c,d)). Low yields of the hybrid in the conditions with low level of stress could be related to other factors which were not included in this study, i.e. field management, pests and diseases or some other issues.

  Table 2 presents statistics for the entire dataset with 2,452 hybrids, where the most of them (1,612) are non-tolerant to any stress or they were not grown in stress conditions. Our methodology classified 680 hybrids as drought tolerant and 55 hybrids as heat tolerant, while 44 hybrids were classified as both heat and drought tolerant but susceptible to combined heat and drought stress. Number of hybrids resistant to all kinds of stress, i.e. heat, drought and stress due to the combination of heat and drought, is 61. Additionally, we investigated the performance of those 61 hybrids in optimal agricultural conditions, considering the yield at the environments with normal irrigation. For each hybrid, we calculated difference between average yield at the environments with normal irrigation and average yield at all other environments. In most of the cases, the difference was positive and only for five hybrids it was negative (see Supplementary Figs. S4 and S5). Also, results of t-test showed that the number of hybrids where there is no statistically significant difference between average yields (we cannot reject the null hypothesis) is 34, while the number of hybrids where we reject the null hypothesis is 27, including those five hybrids with negative yield difference.

**Table 2:** Results of hybrids classification obtained with presented methodology.

Heat resistant	Drought resistant	Heat and drought resistant	Number of hybrids
no	no	no	1612
no	yes	no	680
yes	no	no	55
yes	yes	no	44
yes	yes	yes	61

## Discussion

Development of plant varieties that are resistant to negative impacts of environmental stresses and maintain yield stability are essential to sustain and increase agriculture production^38^. Breeding maize hybrids with stable yield for the drought-prone regions involves integration of multiple technologies that comprise a successful breeding programme^39^. Maize breeders would benefit from accurate models that can predict performance across a range of environmental scenarios^1^. In our approach, it is necessary to have performance of the hybrid across many environments but only for a few years. Hence, this method cuts down time needed to assess if the hybrid is resilient to environmental stress and much faster directs future breeding. Our study started by extensive literature search aimed at selecting weather features suitable for quantifying heat and drought. Also, it introduced new environmental index which is more advanced than one commonly used^40^. The environments were ranked based on the total amount of drought or heat stress present in them, so the performance of hybrids could be examined in a new space defined by the environment ranking. Developed methodology is an example of theory-guided data science^41^, which combined domain knowledge along with machine learning techniques and big data analytics.

This methodology is scalable and it can be applied for any region of the world with proper agricultural data, since soil data are available from SoilGrids^42^, while meteorological data can be retrieved from ERA5, through the Climate Data Store, which is the cornerstone of Copernicus Climate Change Service infrastructure. ERA5 is the fifth generation ECMWF (European Centre for Medium-range Weather Forecast) atmospheric reanalysis of the global climate. Reanalysis combines model data with observations from across the world into a globally complete and consistent dataset using the laws of physics, in the process called data assimilation. Horizontal resolution of hourly data is 0.1° × 0.1°, with the temporal coverage from 1981 to present.

## Supplementary information

Supplementary Information.

## Data Availability

The data underlying this study are third party data available from Syngenta. Syngenta understands and appreciates the need for transparency in research and is ready to make the data available to researchers who meet the criteria for access to confidential data, sign a confidentiality agreement, and agree to work under its supervision. The authors accessed the data through annual Syngenta Crop Challenge in Analytics 2019.
