# Supplementary material for: Engineering Meteorological Features to Select Stress Tolerant Hybrids in Maize
Source: Sci Rep. 2020 Feb 25;10:3421. doi: 10.1038/s41598-020-60366-y (PMC7042286; doi:10.1038/s41598-020-60366-y)
Supplement: Supplementary file 1 — Supplementary Information. [file 41598_2020_60366_MOESM1_ESM.pdf]

Engineering Meteorological Features to Select Stress Tolerant Hybrids in Maize

Gordan Mimić[1,*], Sanja Brdar[1], Milica Brkić[1], Marko Panić[1], Oskar Marko[1], Vladimir Crnojević[1]

[1]University of Novi Sad, BioSense Institute, Novi Sad, 21000, Serbia
gordan.mimic@biosense.rs

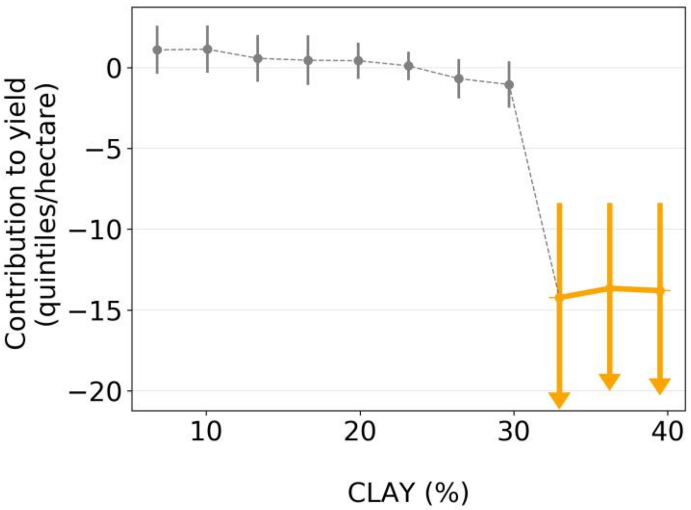

Figure S1. Contribution to the yield for the percentage of clay obtained through the model explanation. Circles represent mean values, bars denote the standard deviation while arrows stand for the significance.

*Corresponding author, E-mail: gordan.mimic@biosense.rs

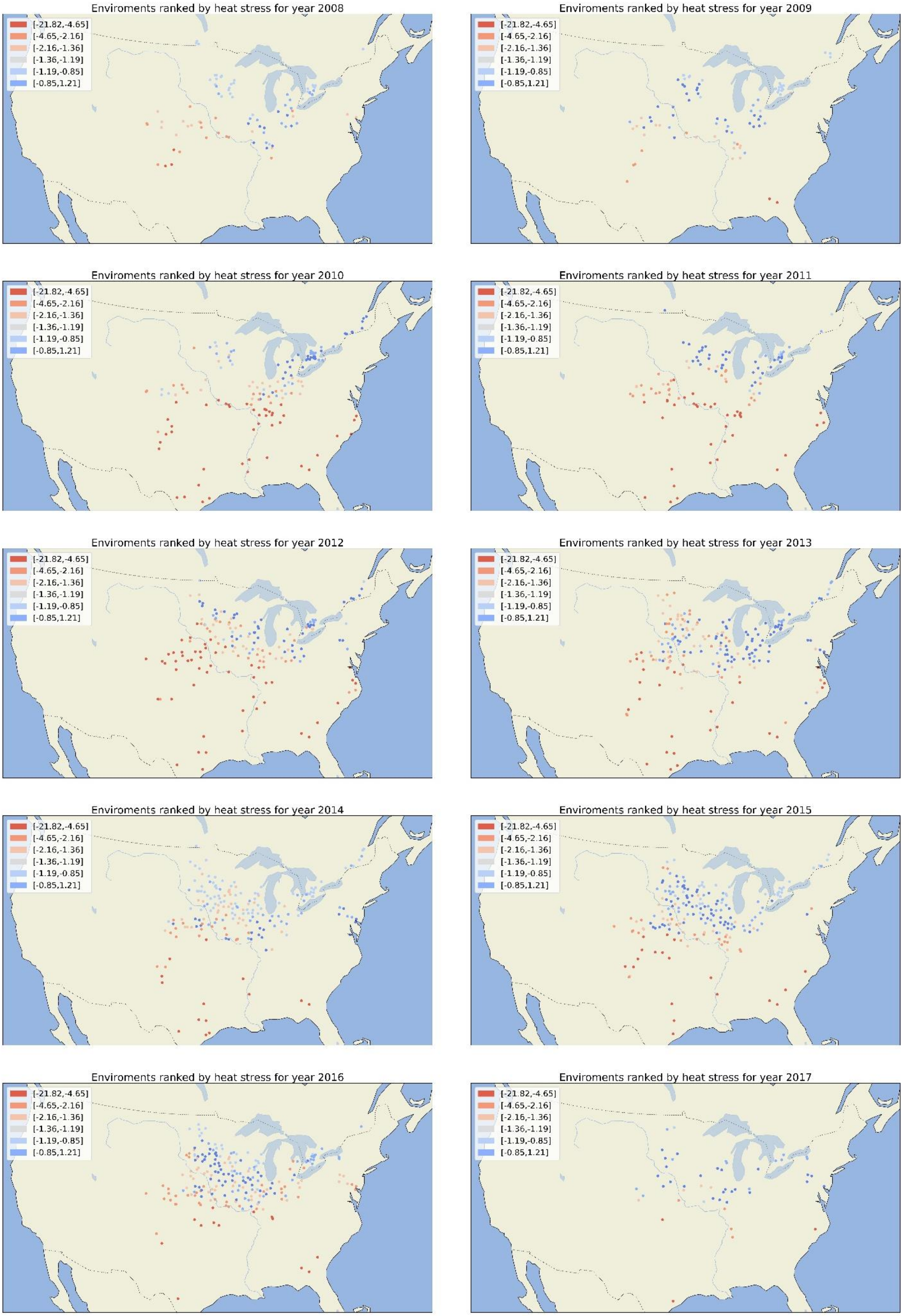

Figure S2. Environments ranked by heat stress for all years used in study.

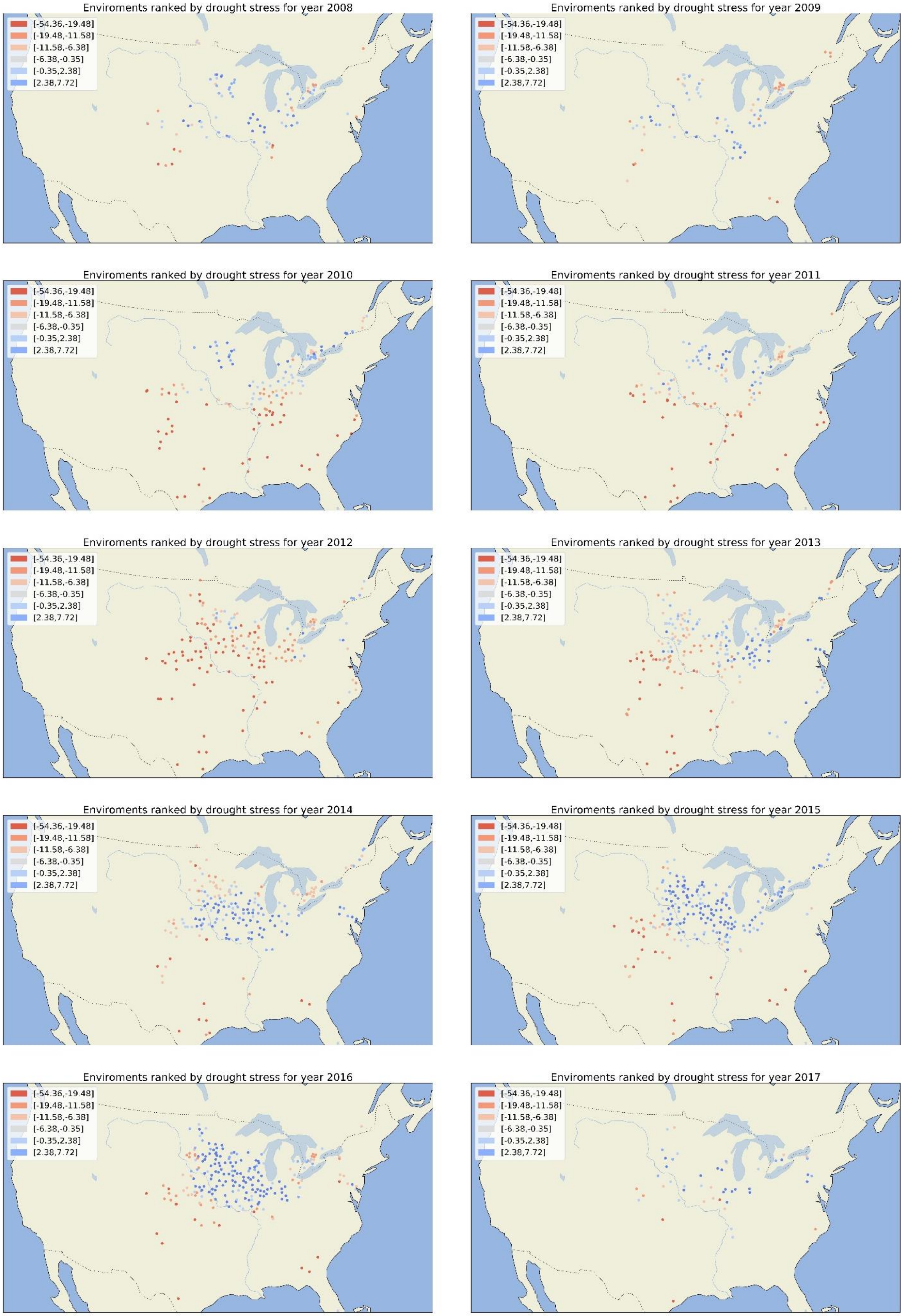

Figure S3. Environments ranked by drought stress for all years used in study.

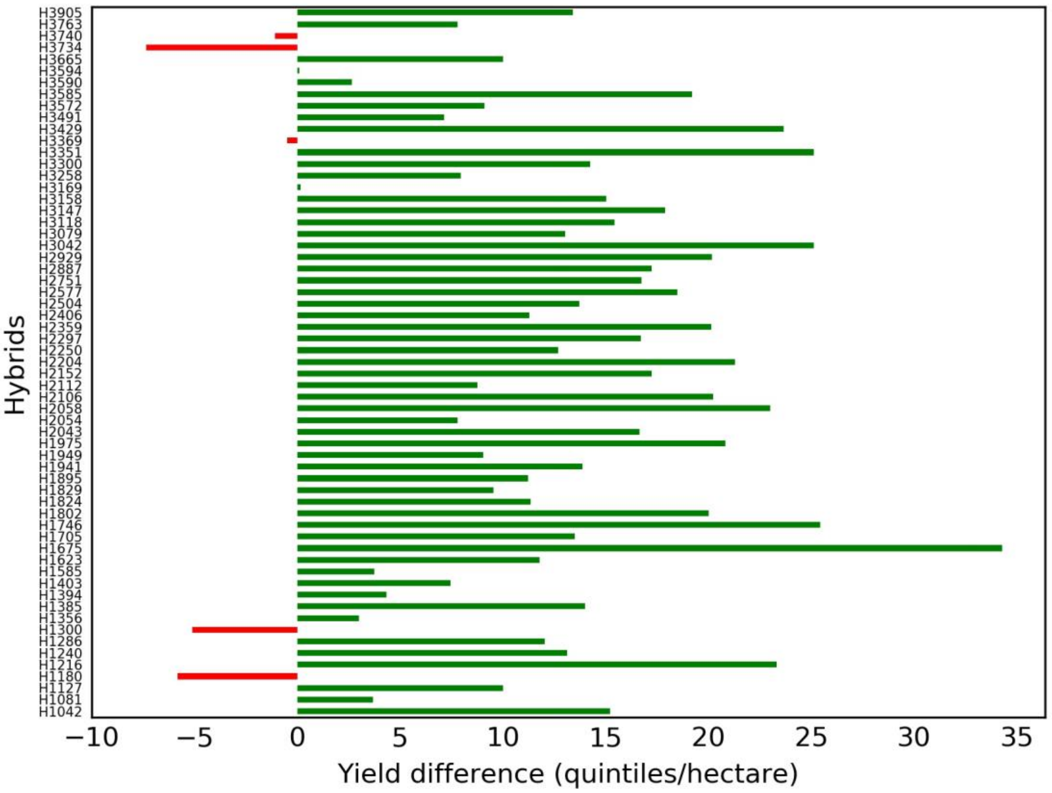

Figure S4. Difference between average yield for the environments with normal irrigation and all other Environments for each of 61 hybrids.

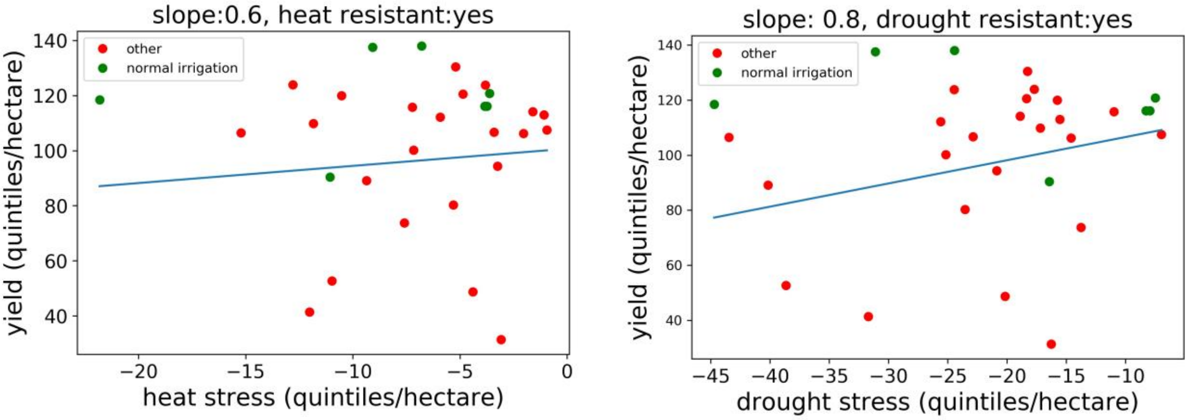

(a) (b)

Figure S5. Performance of hybrid H2058 in different environments with heat stresses (a) and drought stress (b), including those with normal irrigation.
